# Challenging pitfalls in frozen section pathology: a case of mandible ghost cell odontogenic carcinoma and the literature review

**DOI:** 10.1186/s12903-024-04190-0

**Published:** 2024-04-13

**Authors:** Sha-Sha Hu, Jian Yang, Hai-Fei Zhang, Jie Chen, Xin-Nian Li, Fu-Jin Liu, Bo Wang

**Affiliations:** 1 Department of Pathology, Hainan General Hospital (Hainan Affiliated Hospital of Hainan Medical University), Haikou, 570311 China; 2 Department of Wound Repair, The First Affiliated Hospital of Hainan Medical University, Hainan Medical University, Haikou, 570311 China

**Keywords:** Ghost cell odontogenic carcinoma, Misdiagnosis, Frozen section pathology, Squamous cell carcinoma, Case report

## Abstract

**Background:**

Ghost cell odontogenic carcinoma (GCOC) is a rare malignancy characterized by the presence of ghost cells, preferably in the maxilla. Only slightly more than 50 case reports of GCOC have been documented to date. Due to the rarity of this tumor and its nonspecific clinical criteria, there is a heightened risk of misdiagnosis in clinical examination, imaging findings, and pathology interpretation.

**Case presentation:**

A 50-year-old male patient presented to the hospital due to experiencing pain in his lower front teeth while eating for the past 2 months. Upon examination, a red, hard, painless mass was found in his left lower jaw, measuring approximately 4.0 cm × 3.5 cm. Based on the malignant histological morphology of the tumor and the abundant red-stained keratinized material, the preoperative frozen section pathology misdiagnosed it as squamous cell carcinoma (SCC). The surgical resection specimen pathology via paraffin section revealed that the tumor was characterized by round-like epithelial islands within the fibrous interstitium, accompanied by a large number of ghost cells and some dysplastic dentin with infiltrative growth. The malignant components displayed marked heterogeneity and mitotic activity. Additionally, a calcified cystic tumor component of odontogenic origin was observed. Hemorrhage, necrosis, and calcifications were present, with a foreign body reaction around ghost cells. Immunoreactivity for β-catenin showed strong nuclear positivity in tumor cells, while immunostaining was completely negative for p53. The Ki67 proliferation index was approximately 30–40%. The tumor cells exhibited diffuse CK5/6, p63, and p40 immunoreactivity, with varying immunopositivity for EMA. Furthermore, no *BRAF*^*V600E*^ mutation was identified by ARMS-PCR. The final pathology confirmed that the tumor was a mandible GCOC.

**Conclusion:**

We have reported and summarized for the first time the specific manifestations of GCOC in frozen section pathology and possible pitfalls in misdiagnosis. We also reviewed and summarized the etiology, pathological features, molecular characteristics, differential diagnosis, imaging features, and current main treatment options for GCOC. Due to its rarity, the diagnosis and treatment of this disease still face certain challenges. A correct understanding of the pathological morphology of GCOC, distinguishing the ghost cells and the secondary stromal reaction around them, is crucial for reducing misdiagnosis rates.

## Background

Ghost cell odontogenic carcinoma (GCOC) is a very rare malignancy originating from odontogenic epithelium, typically affecting patients aged from 40 to 70 years, with a higher occurrence in males [1]. GCOC, characterized by poorly demarcated lesion radiologically, ameloblastoma-like epithelium, prominent ghost cells and cytological evidence of malignancy, is about the rarest of the ghost cell lesions, accounting for approximately 0.23% of all odontogenic tumors and less than 3% of all ghost cell lesions [1–3]. In 2005, it was included in the World Health Organization (WHO) classification of malignant odontogenic tumors [4]. Since Ikemura et al. firstly recorded a case in detail in 1985 [5], only slightly more than 50 case reports of GCOC have been documented to date [6]. About 55% cases of GCOC are thought to originate from de novo, others arise from pre-existing calcifying odontogenic cyst (COC) or dentinogenic ghost cell tumor (DGCT) [7]. These three tumors manifest similar clinical and radiological features, making the diagnosis challenging. Given the rarity and nonspecific clinical criteria of the tumor, clinical examination, imaging findings and pathology are also prone to misdiagnosis.

Here, we report a rare case of mandible ghost cell odontogenic carcinoma that was misdiagnosed as squamous cell carcinoma on intraoperative incisional biopsy frozen section pathology.

## Case presentation

**Fig. 1:**
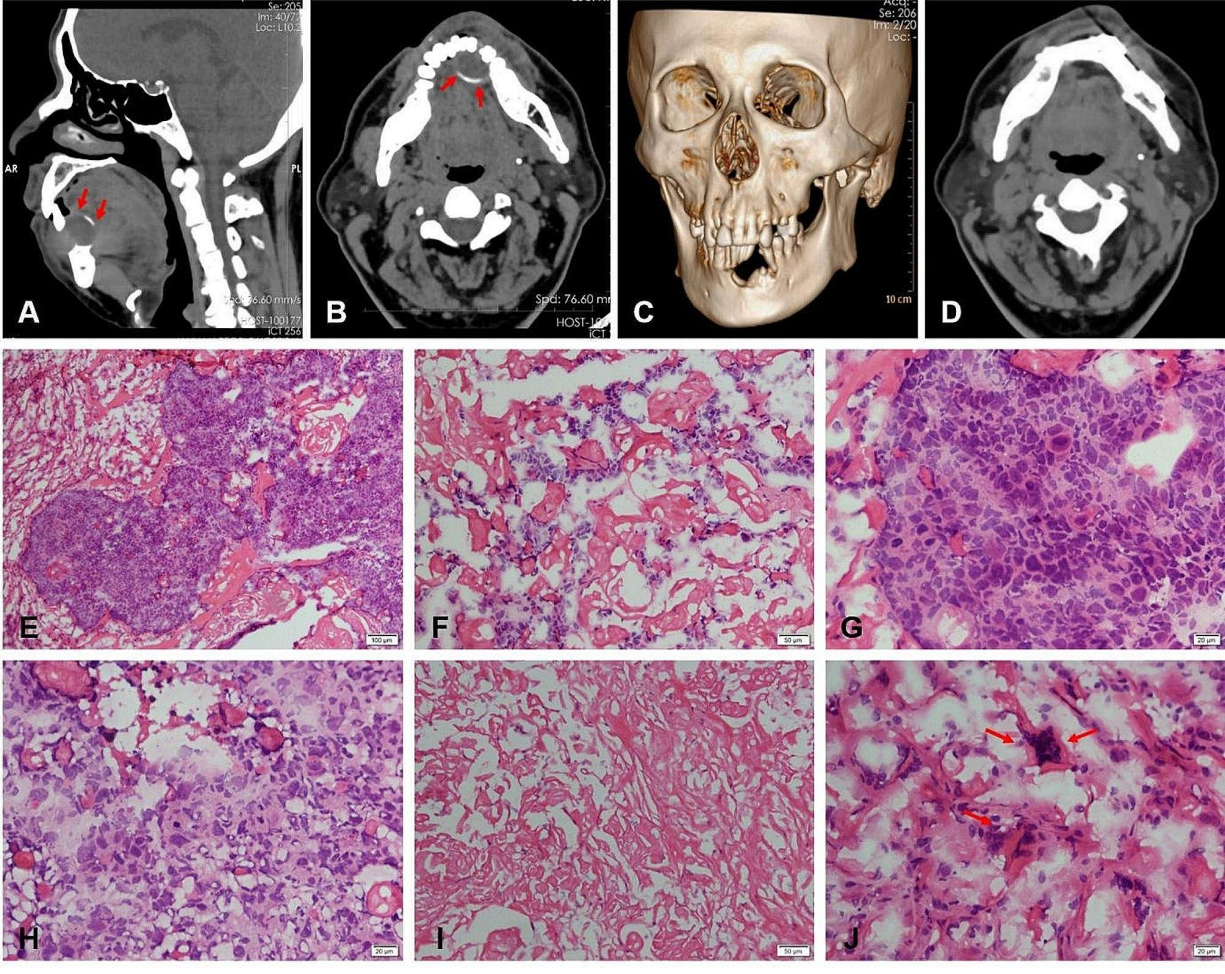
Preoperative CT and intraoperative pathologic frozen examination. (**A**, **B**) Computed tomography (CT) showed a soft tissue mass with bone destruction in the mandible (red arrows). (**C**) 3D reconstruction of CT scan showed significant loss in the median alveolar region of the mandible with no signs of fracture (**D**) Postoperative images. (**E**, **F**) In frozen section pathology, resected tumor showed variable patterns of solid nests or cords. (**G**, **H**) The tumor cells with pleomorphism, increased N/C ratio, nuclear hyperchromatism, and mitotic activity. (**I**) There were large numbers of homogeneous red-stained unstructured or hyaline stroma similar to keratinization. (**J**) Foreign body granuloma reaction could be seen in the surrounding interstitium (red arrows).

In April 2021, a 50-year-old male patient presented to the hospital with complaints of painful feeling while eating in his lower anterior teeth for 2 months. His examination revealed a red, hard, painless swelling of approximately 4.0 cm × 3.5 cm in size, located in the left mandible. The patient exhibited poor overall oral hygiene, and his teeth had grade II mobility with caries. However, no enlargement of the lymph nodes in the lower jaw or the oral cavity was detected. The rest of the dental specialty examination revealed no abnormalities. This patient had a long-term betel nut chewing habit and no other genetic or chronic diseases. The attending physician at the time diagnosed a mandibular cyst. Maxillofacial computerized tomogram (CT) suggested a soft tissue mass with bone destruction in the median alveolar region of the mandible (Fig. 1A-C), which was considered a tumorigenic lesion. Postoperative CT showed no residual mass (Fig. 1D).

The initial frozen biopsy section examination displayed the lesion dominated by large numbers of homogeneous red-stained unstructured or hyaline stroma, resembling keratinization (Fig. 1I). Scattered among these stromal elements were tumor cells exhibiting pleomorphism, increased nuclear/cytoplasmic (N/C) ratio, nuclear hyperchromatism, and mitotic activity (Fig. 1G, H). The tumor cells formed solid nests or cords (Fig. 1E, F), indicative of a malignant epithelial tumor. Additionally, a foreign body granuloma reaction was observed in the surrounding interstitium (Fig. 1J). In conclusion, the final frozen section pathology diagnosis was SCC.

Upon admission, the patient was initially suspected to have a mandibular cyst. However, intraoperative freezing indicated the presence of squamous cell carcinoma in the mandibular mass. Considering the significant shift in the tumor’s nature, the medical team made the decision to modify the initially planned surgical approach and broaden the extent of the procedure after consulting with the patient’s family. The revised surgical approach consisted of several steps. First, a partial resection of the mandible and the mass was performed. This was followed by bilateral cervical lymphadenectomy to remove any potentially affected lymph nodes. The next step involved repairing the defect in the fundus. To reconstruct the area, an excision was performed, and a vascularized free peroneal myocutaneous flap was used as a graft. A small arterial anastomosis was then carried out to ensure proper blood supply to the graft. Solid internal fixation was applied to stabilize the mandible. Additionally, the fibula, along with its blood vessels, was extracted for further reconstruction purposes. Finally, a tracheotomy was performed. These modifications to the surgical procedure were made in order to effectively address the presence of squamous cell carcinoma and ensure the best possible outcome for the patient.

**Fig. 2:**
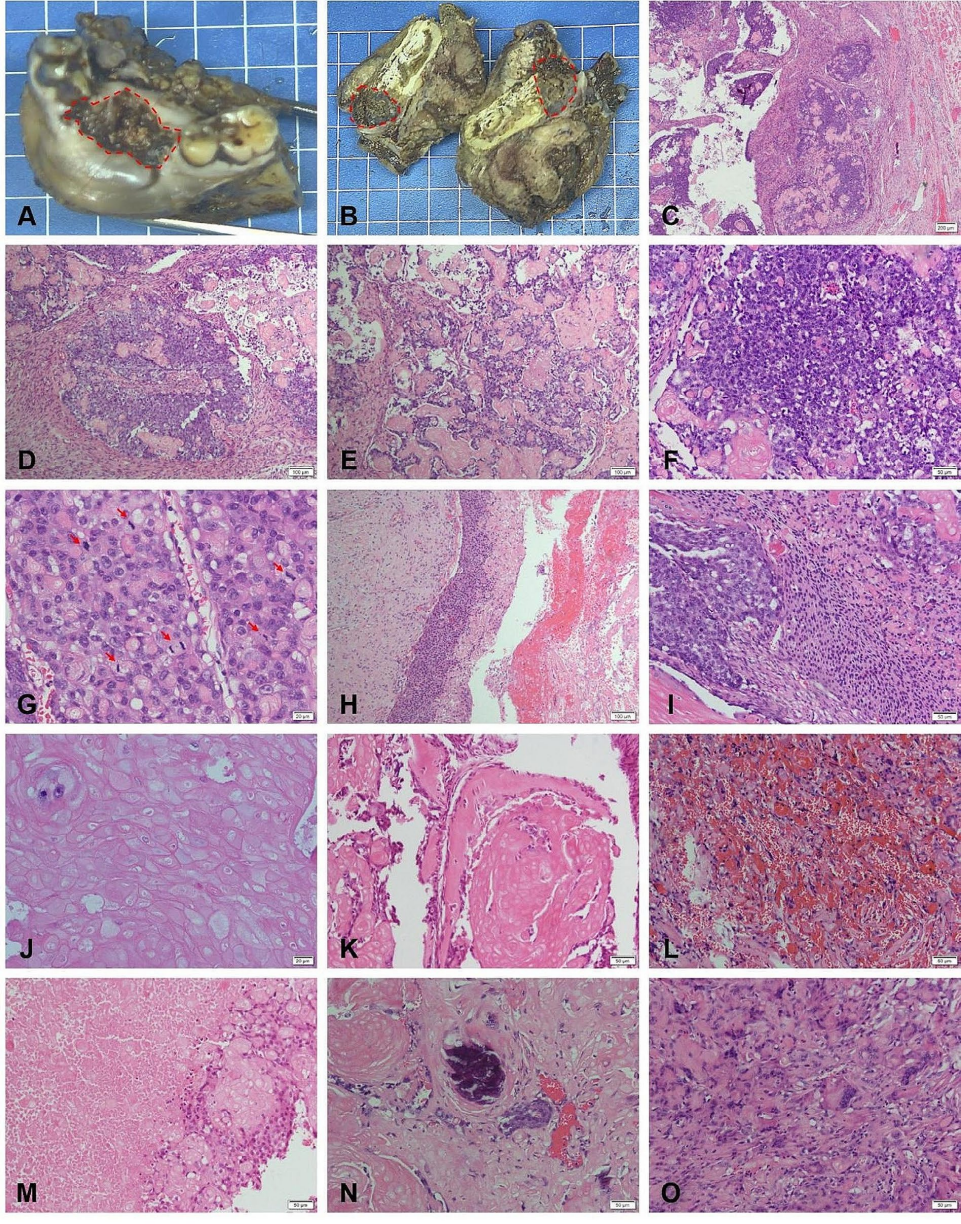
Postoperative pathology analysis. (**A**, **B**) Gross excision specimen and tumor incision surface (red circles). (**C**) Microscopically, paraffin section showed the tumor boundary was not clear. (**D**, **E**) Hematoxylin and eosin stain (HE) showed that tumor cells are arranged in solid nests and anastomosing cords. (**F**, **G**) The tumor was composed of small cells with hyperchromatic nuclei or large cells with vacuolated nuclei, with marked heterogeneity and mitotic activity (red arrows). (**H**, **I**) A calcified cystic tumor component of odontogenic origin could be seen and the malignant epithelial component were separated from or mixed with the benign lesion. (J-O) Ghost cells (**J**), dentinoid material (**K**), hemorrhage (**L**), necrosis (**M**) and calcifications (**N**) could be found, with foreign body reaction around ghost cell (**O**).

The treatment involved excision of part of the mandibular bone and mass. Grossly, surgical specimen measured 6.5 × 5.5 × 4.0 cm, with 5 teeth attached to it. The area of alveolar mucosa showed an ulcerated mass (Fig. 2A). The cut surface of the tumor was 2.2 × 1.3 × 2.1 cm in diameter, presented as a gray to taupe solid mass with areas of hemorrhage and cystic change and invaded the mandible (Fig. 2B).

The histological examination revealed the tumor was characterized by round-like epithelial islands within the fibrous interstitium, accompanied by a large numbers of ghost cells and a little dysplastic dentin with infiltrative growth (Fig. 2C). Histopathological sections revealed solid nests and anastomosing cords (Fig. 2D, E). The malignant components consisted of round-like epithelial islands, with some cells appearing as small round cells with deeply stained nuclei, while others exhibited large cells with vacuolated nuclei, displaying marked heterogeneity and mitotic activity (Fig. 2F, G). A calcified cystic tumor component of odontogenic origin could be seen. The malignant epithelial component was observed either separated from or mixed with the benign lesion (Fig. 2H, I). The ghost cells were round or ovoid, with red-stained cytoplasm, disappearing uncolored nuclei, and empty bright areas at the nuclei (Fig. 2J). Dentinoid material and hemorrhage, necrosis and calcifications could be found, with foreign body reaction around ghost cell (Fig. 2K-O).

**Fig. 3:**
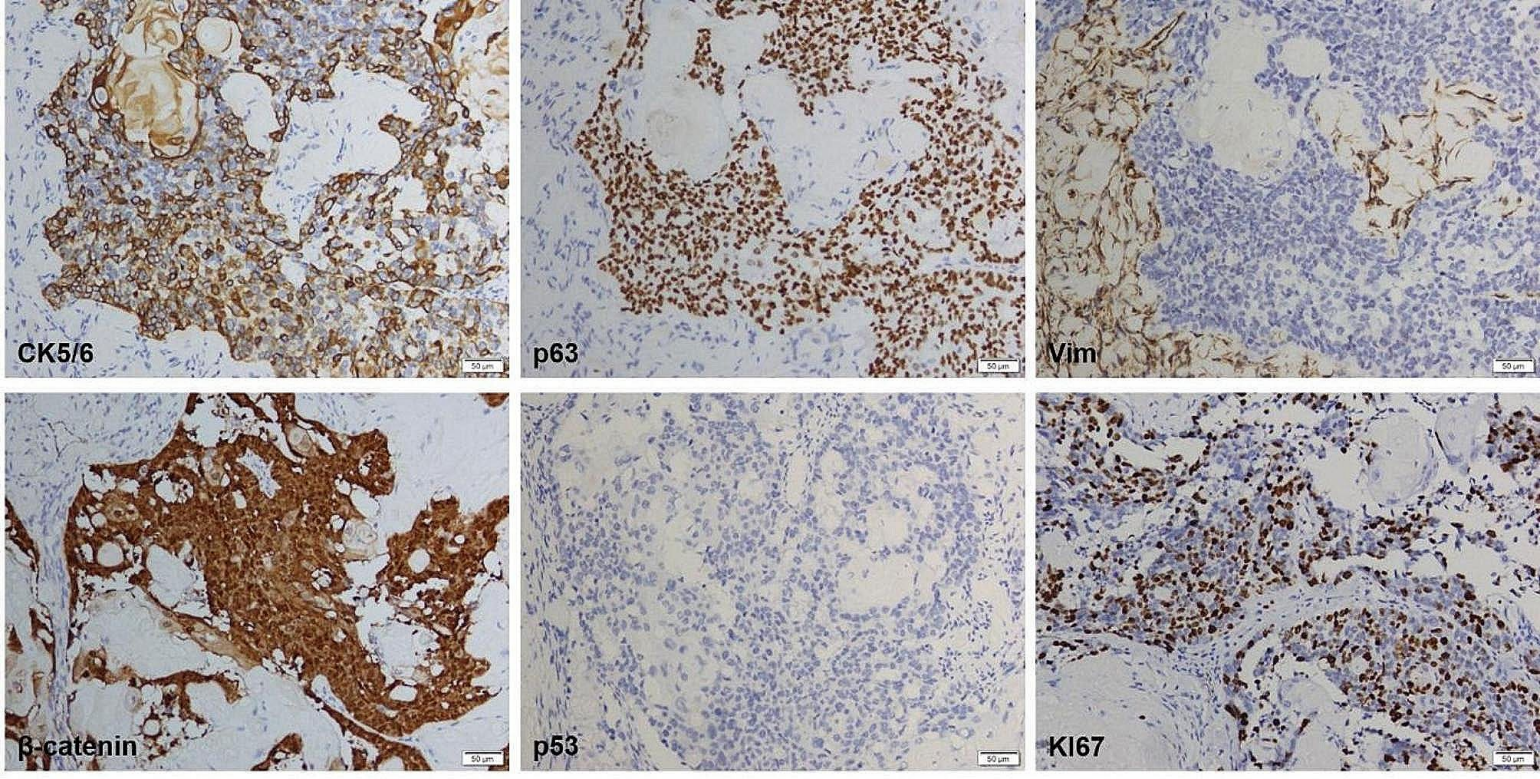
Immunohistochemistry showed expression of CK5/6, p63, Vim, β-catenin, p53 and Ki67.

An extensive immunohistochemical panel was performed. Immunoreactivity for β-catenin showed strong nuclear positivity in tumor cells. Immunostaining was completely negative for p53. The Ki67 proliferation index was around 30–40%. The tumor cells showed diffuse CK5/6, p63 and p40 immunoreactivity. There was varying immunopositivity for EMA. Immunostaining was negative for Vimentin (Vim), S-100, Synaptophysin (Syn) and Chromogranin A (CgA) (Fig. 3). No BRAF V600E mutation was identified by amplification refractory mutation system polymerase chain reaction (ARMS-PCR).

**Fig. 4:**
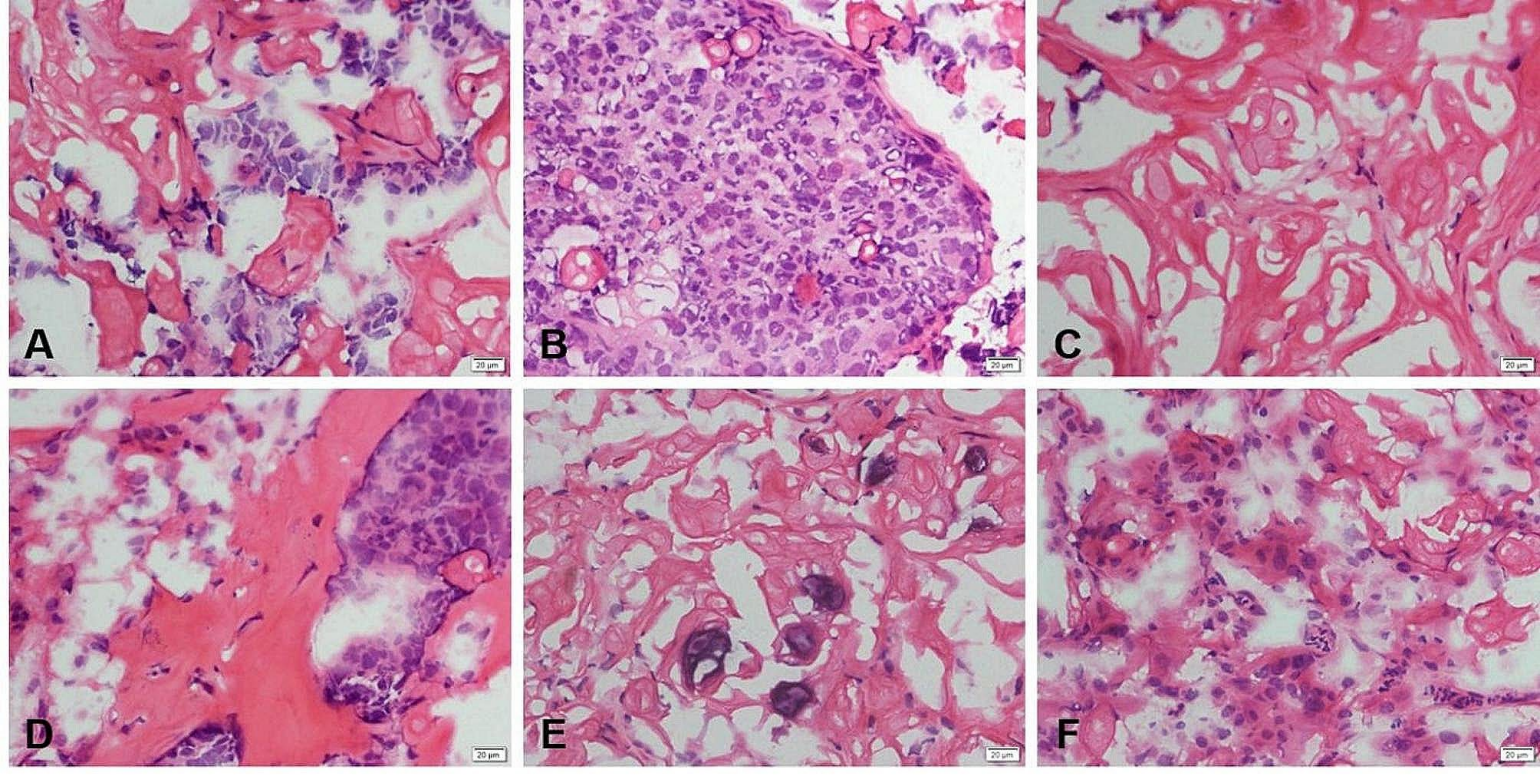
The possible pitfalls of frozen section pathology diagnosis of GCOC. (**A**) A large number of ghost cells could be seen, (**B**) There was no intercellular bridge between cells. (**C**) The homogeneous red-stained unstructured materials were very characteristic ghost cells. (**D**-**E**) A little dentin material (**D**), calcifications (**E**) and foreign body granuloma reaction around ghost cells (**F**) could be found by careful observation.

The final pathology via paraffin section showed that the tumor was a mandible GCOC. We reviewed and summarized the possible pitfalls of frozen section pathology diagnosis of GCOC. A large numbers of ghost cells could be seen between the tumor cells of GCOC, and there was no intercellular bridge between cells (Fig. 4A, B). The homogeneous red-stained unstructured materials were very characteristic ghost cells (Fig. 4C), and a little dentin material could be found by careful observation (Fig. 4D). In addition, calcifications (Fig. 4E) and foreign body granuloma reaction (Fig. 4F) around ghost cells were also suggestive for the diagnosis of GCOC.

The patient finally recovered and was discharged in May 2021. Under the strict regular physical and imaging examinations, the patient has no signs of tumor recurrence within two years of follow-up. Patients gave their affirmation and adequate cooperation to the process and results of treatment.

## Discussion

This report described a rare case of mandibular GCOC that was misdiagnosed as SCC by frozen section pathology during the perioperative period. Previous examinations and imaging findings were inconclusive. Intraoperative frozen section pathology was diagnosed as SCC on the basis of cytological evidence of malignancy and a large number of keratin-like red-stained unstructured structures. Until the final paraffin section pathology corrects the diagnosis as GCOC.

GCOC is an extremely rare destructive and aggressive malignant odontogenic tumor. Due to its different histopathological features, various terms have been used to define the disease, including calcifying ghost cell odontogenic carcinoma, aggressive epithelial ghost cell odontogenic tumors, malignant epithelial odontogenic ghost cell tumor, carcinoma arising in a calcifying odontogenic cyst, malignant calcifying ghost cell odontogenic tumors and malignant calcifying odontogenic cyst [8, 9]. So far, only more than 50 cases have been reported in the English literature, with about 40% of the cases occurring in Asian patients [6]. GCOC has a male predominance, occurring in individuals from 3 to 92 years [10]. In one study, it was suggested that GCOC was twice as common in the maxilla as in the mandible [8]. Another statistical survey showed that GCOC occurred more frequently in the maxilla than in the mandible, with 31 out of 51 patients occurring in the maxilla [11]. These tumors have been intraosseous and mandibular lesions are usually in the molar area [12]. Given the rarity of the disease and the non-specificity of its clinical features, little is known about GCOC. Therefore, the progression of GCOC is unpredictable. Meanwhile, it may vary from slow progression to rapid destructive behavior, with recurrence and occasional distant metastasis to axillary skin, brain, and lung [13].

The diagnosis of GCOC is challenging and difficult for the first attending physician, and even pathologists face a high risk of misdiagnosis due to its rarity, complexity and inexperience. In imaging, GCOC does not have specific imaging features, so pathological testing remains the primary and most important way to identify. For the differential diagnosis of GCOC, the main differentiators are benign odontogenic tumors, dentinogenic ghost cell tumor (DGCT), calcifying odontogenic cyst/ calcifying cystic odontogenic tumor (COC/ CCOT), odontomas, cholesterol granuloma of the maxillary sinus (CGMS), amelobalstoma and also in craniopharyngiomas and pilomatricomas [14]. In addition, if ghost cells are not present in the frozen section, the possibility of ameloblastic carcinoma should also be considered. Generally benign lesions tend to have well-defined margins, while malignant tumors are mostly destructive and ill-defined [15]. WHO describes the DGCT parenchyma as presenting an ameloblastomatous proliferation with occasional significant component of hyperchromatic basaloid cells [16]. Exuberant areas with spindle-shaped cells and sieve-like structures can also be observed in some cases [17]. However, one must be alert to the fact that while ameloblastomas and sieve patterns can be found in other odontogenic lesions such as ameloblastomas and adenoid ameloblastomas, these lesions may also have scattered ghost cells [18]. CGMS is characterized by a large number of cholesterol clefts surrounded by multinucleated giant cells, histiocytes, plasma cells, lymphocytes, and hemosiderin deposits [19, 20]. CCOT is characterized by proliferation of odontogenic epithelium and scattered nests of ghost cells and calcifications that may form the lining of a cyst, or present as a solid mass [21]. COCs are recognized by cystic proliferation with a fibrous capsule. The thickness of lining epithelium may vary between 4 and 10 layers. Areas of calcification and ghost cells can be observed [22].

In our case, the frozen section pathology was misdiagnosed as SCC. If the pathologist has insufficient diagnostic experience and encounters challenges like easy deformation and poor staining of frozen pathological sections, there is a risk of mistaking ghost cells for keratinized cells without careful identification. Therefore, distinguishing GCOC from well-differentiated SCC is crucial. Reviewing our cases and the pathological features of the two tumors, we summarize the following points of differentiation: (1) GCOC is sometimes secondary to COC or DGCT with mixed or segregated benign epithelial components and malignant epithelial components, which can be seen in our paraffin section pathology; whereas SCC can be seen with varying degrees of squamous intraepithelial lesions. (2) GCOC shows ameloblastoma-like epithelium with fenestrated peripheral cells in the cell nest, a typical structure not seen in our case, while basal cell-like SCC shows fenestrated peripheral cells in the cell nest. (3) In GCOC, a large numbers of ghost cells were seen around the nest and anastomotic strips, i.e., round or ovoid cells with red-stained cytoplasm and absent, uncolored nuclei, and empty bright areas in the nuclei; a large number of keratinized cells were seen in the center of the nest of well-differentiated SCC cells and formed keratinized beads. GCOC keratinization differs from normal keratinization in several aspects. Firstly, GCOCs are larger than keratotic squames. Secondly, they are often vacuolated, containing small fluid-filled spaces. Lastly, GCOCs exhibit prominent remnants of the nuclear membrane [23]. Failure to correctly identify ghost cells is also a major cause of misdiagnosis in our frozen section pathology. (4) GCOC consists of small cells with deeply stained nuclei or large cells with vacuolated nuclei and basophilic cells; well-differentiated SCC cells are large with eosinophilic or biphilic cytoplasm and intercellular bridges are seen. (5) In addition, consistent with what has been reported in other literature, the presence of dentin as well as calcification and foreign body granulomatous reaction in the ghost cell area can help to identify GCOC [10, 23], and after careful observation, these lesions are seen in our frozen pathological sections.

The etiology of GCOC is controversial, and current pathogenesis theories include: GCOC occurs secondary to calcifying cystic odontogenic tumors [24]; GCOC is caused by dentinogenic ghost cell tumors [20]; de novo, with no previous associated lesions [25, 26]; genetic mutations are a possible direction [7]. Rappaport et al. reported that mutation of the β-catenin gene was noted at codon 33 in GCOC [27]. Three other genomic alterations in GCOC: CTNNB1 S33C, CREBBP K1741* and MLL2 S1997fs*44 [27]. An extensive integrative genomic and transcriptomic analysis of GCOC studied by Bose et al. reported numerous genomic alterations [28]. P53 overexpression and UBR5 mutations were also reported in the GCOC [29], while in another study genetic abnormalities were found in NOTCH1 and PTEN due to deletion [18]. However, one must be alert to the fact that while ameloblastomatous and cribriform patterns can be found in other odontogenic lesions such as ameloblastoma and adenoid ameloblastoma, these lesions may also have scattered ghost cells [16, 30]. In our case, immunohistochemical results showed a positive β-catenin diffuse nucleus with complete deletion of p53 expression and showed a high proliferation index of ki-67. We used ARMS-PCR to detect *BRAF*^*V600E*^, but the test result was negative.

Due to the extreme scarcity of cases, there is still considerable disagreement among different authors regarding the prevalent location of GCOC, its metastatic characteristics, and treatment options. Currently, more researchers believe that the main site of predilection for GCOC is the maxilla, and the most common clinical symptom is a painful swelling of the upper jaw accompanied by local sensory abnormalities [31]. The most typical radiological features of GCOC show a mixed pattern of radiolucent and radiopaque lesions with ill-defined borders, with or without root resorption and tooth displacement [31, 32]. However, in this case, the boundaries of GCOC are well defined, and the rare morbidity and atypical imaging pattern are more likely to lead to an error in the initial clinical diagnosis, making the pathological diagnosis of GCOC extremely important. The current GCOC recommended treatment is extensive surgical excision of at least 5 mm of free margin with no residual outside the incision margin [33]. The most frequent procedures include marginal, segmental or partial resection or total maxillectomy, depending on the size of the lesions [32, 34]. Post-surgical treatment options include adjuvant chemotherapy, adjuvant immunotherapy, and adjuvant radiotherapy, but the effectiveness of treatment remains controversial to this day. Qin Y, et al. reported significant symptom improvement in patient who underwent extensive surgery followed by two cycles of chemotherapy and radiotherapy, along with four rounds of weekly chemotherapy [20]. However, some researchers have pointed out that the benefit of adjuvant radiotherapy for GCOC patients is difficult to determine [24, 26]. In 2015, Ahmed et al. reported the first case treated successfully with aggressive multimodal therapy in a 10 year old patient with regional lymph node metastasis that included surgery, adjuvant chemoradiation, and adjuvant immunotherapy [35]. Lu Y et al. reported that the 5-year survival rate of GCOC was about 73% [36]. In another related paper, the recurrence rate for recurrence, metastasis, and survival in GCOC was reported to be 63.4% [32]. Due to the limited number of cases and high recurrence rate, our knowledge of GCOC is limited, and prognosis is difficult to predict. Therefore, long-term follow-up and monitoring are necessary.

## Conclusions

We report a highly unusual case of GCOC, initially misdiagnosed as SCC on frozen section pathology, and subsequently diagnosed as GCOC through a series of pathologic examinations. GCOC has been poorly studied due to its nonspecific clinical features and extremely low incidence, especially since frozen section pathology reports have never been reported. At the same time, we summarized the clinical features, imaging characteristics and treatment options of GCOC. Our report presents, for the first time, the pathological presentation of GCOC through frozen section pathology, along with a thorough analysis of potential misdiagnosis pitfalls for more pathologists, in order to deepen their understanding of this disease and reduce the misdiagnosis rate of intraoperative freezing. This case provides valuable and informative data and insights, contributing to our understanding of this rare entity with limited reported cases.

## Data Availability

All data generated or analysed during this study are included in this published article.

## Abbreviations

GCOC: Ghost cell odontogenic carcinoma
SCC: squamous cell carcinoma
CCOT: calcifying cystic odontogenic tumor
COC: calcifying odontogenic cyst
DGCT: dentinogenic ghost cell tumor
CT: computerized tomogram
ARMS-PCR: amplification refractory mutation system polymerase chain reaction
